# The burden of chronic kidney disease attributable to high sodium intake: a longitudinal study in 1990–2019 in China

**DOI:** 10.3389/fnut.2024.1531358

**Published:** 2025-01-15

**Authors:** Yongyao Shen, Liying Jiang, Jin Yu, Bo Chen, Aidong Liu, Yongjin Guo

**Affiliations:** ^1^Graduate School, Shanghai University of Traditional Chinese Medicine, Shanghai, China; ^2^Jiading Central Hospital, Shanghai University of Medicine & Health Sciences, Shanghai, China; ^3^College of Public health, Shanghai University of Medicine & Health Sciences, Shanghai, China; ^4^Department of Epidemiology, School of Public Health, Nantong University, Nantong, Jiangsu, China; ^5^National Institute for Nutrition and Health, Chinese Center for Disease Control and Prevention, Beijing, China

**Keywords:** chronic kidney disease, high sodium intake, disease burden, China, Global Burden of Disease (GBD)

## Abstract

**Objective:**

Elevated sodium consumption is associated with increased risk for chronic kidney disease (CKD) and data for this disease burden attributable to high sodium intake in China from 1990 to 2019 are scarce in China. Our study aims to estimate and present the national burden of CKD attributable to high sodium intake, as well as its profile.

**Methods:**

The regional disease burden data from the China Center for Food Safety Risk Assessment (CFSA) from 1990 to 2019 were compiled based on the methodology of GBD 2019. CKD burden [deaths and disability-adjusted life years (DALYs)] was quantified according to population group (age, gender) and regions categories (province, SDI). The estimated annual percentage change (EAPC) in age-standardized mortality rate (ASMR) and age-standardized DALYs rate (ASDR) were calculated to describe long-term trends.

**Results:**

Totally, the number of deaths of CKD attributable to high sodium intake reached 95,000, with DALYs amounting to 2.59 million person-years in 2019, while the trends of ASMR (EAPC: -0.31, 95%CI: −0.46, −0.17%) and ASDR (−0.33, 95%CI: −0.48, −0.18%) were down during the observation period. The age-specific numbers and rates of deaths, as well as DALYs increase with age are higher in males than in females. Significant disparities in CKD burden attributable to high sodium intake were observed across provinces and SDI regions. In both 1990 and 2019, the number of deaths and DALYs were higher in middle SDI regions, while low-middle SDI regions had highest ASMR and ASDR. The EAPC of ASDR was found to be significantly negatively correlated with the 1990 ASDR (*ρ* = −0.393, *p* = 0.024), and the EAPC of ASMR and ASDR were also significantly negatively correlated with the 2019 SDI (ASMR:ρ = −0.571, *p* < 0.001; ASDR:ρ = −0.368, *p* = 0.035).

**Conclusion:**

High sodium intake accounted for a sizeable burden of disease from 1990 to 2019 in China, also presents sexual and geographic variations. Despite a slight decreasing trend exists, the absolute number increased as much as twofold, particularly among males and seniors. Targeting to reduce sodium intake remains a priority, and progress requires systematic monitoring and evaluation, particularly in middle SDI regions that are experiencing rising trends and low-middle SDI regions being susceptible to approaches.

## 1 Introduction

Chronic kidney disease (CKD), defined as the presence of persistent abnormalities of renal structure and function for a period exceeding 3 months, is a major public health concern that affects approximately 697 million cases globally, emerging as leading causes of death and disability with over 40 million disability-adjusted life-years (DALYs) and 1.4 million deaths (1, 2). China has the largest number of CKD patients, with 132 million cases in 2017, accounting for 20% of the total number (3). CKD has presented an increasing social, health and economic burden worldwide. The aging Chinese population is predicted to contribute to continuous increase in prevalence.

CKD could generally progress to end-stage renal disease (ESRD) when it is not timely diagnosed and finely treated in a proper manner. Most importantly, patients with ESRD have significant mortality risk and bear a considerable financial burden for the treatment. The financial burden of hospitalization for CKD in China constituted 6.3% of the total medical cost in 2015, amounting to approximately 3.43 billion dollars, and the annual cost in United States is approximately 49 billion dollars (4, 5). Although common, CKD can be clinically silent, and the cost of the disease will burden the healthcare system.

CKD encompasses at least one of the following: (1) Glomerular filtration rate (GFR) less than 60 mL/min/1.73 m^2^; (2) albuminuria (i.e., urinary albumin concentration ≥ 30 mg/24 h or urinary albumin-to-creatinine ratio [ACR] ≥ 30 mg/g); (3) abnormalities of the urinary sediment, histology, or imaging suggestive of renal damage; (4) renal tubular disease; or (5) history of renal transplantation (6). Identified modifiable risk factors associated with CKD include hypertension, diabetes, obesity, smoking and poor dietary habits (7–10). Elevated sodium consumption is associated with increased blood pressure, an identified risk factor for the development and progression of CKD (11). High sodium intake was initially reported to be related with CKD in 1949, while low sodium intake represents a beneficial factor for CKD development (12). Despite low sodium intake is associated with an increased risk of mortality for CKD patients, the role of high sodium intake as a potential trigger for CKD has been recognized and serves as a contributor to the progression to end-stage renal disease (13, 14). The high-sodium-diet causes impaired excretion of sodium and accumulation of sodium in tissues in patients with renal failure, which in turn leads to an increase of blood pressure (15). Moreover, high sodium intake has been linked to an increased prevalence of proteinuria, and it is a significant predictor of the prognosis of CKD (11). Both hypertension and increased proteinuria have been regarded as key risk factors for renal events. Similarly, high sodium intake has detrimental impact on the development and prognosis of CKD through the induction of an inflammatory response and fluid retention, exacerbating the progression of CKD patients (16). Therefore, maintaining a reasonable sodium intake is of particular importance for the special population with CKD.

The World Health Organization (WHO) recommends that adults should limit their sodium intake to a maximum of 2 g or 87 mmol per day (17), most dietary guidelines recommend a daily sodium intake of no more than 100 mmol (2,300 mg) for patients with CKD. Notwithstanding the widespread adoption of measures to control sodium intake, it remains challenging to maintain sodium intake at or below the recommended level. The global average sodium intake has amounted to 4,310 mg/d for individuals and estimates for CKD patients has reached 150–200 mmol/d (18), which significantly exceed the recommended level. Given the greater amount of condiment use in Chinese cuisine, sodium intake in Chinese population is involved in the concern at the national level. The results of the 2012 Chinese Population Health Status Survey indicated that the average daily sodium intake of adults was 5,013 mg (19), exceeding the recommended level as much as 25%.

Even where results of large-scale CKD screening programs are available, data sources report CKD estimates only for selected populations (limited by age group, geography, occupation, etc). There are no data for CKD epidemiology for many regions across China. Notably, the dynamics of CKD burden remain unclear primarily because of the continuous development and the variations in spatial distribution. The study is to delineate the trajectory of the CKD disease burden attributable to high sodium intake in China from 1990 to 2019. Our study is a major effort to collect and incorporate into one system all available data for CKD diseases and apply comprehensive statistical modeling to produce comparable estimates of the burden at the national level, and further give more clarity on prevention strategies for CKD patients in the primary care setting with the ultimate goal of reducing the health and economic burdens.

## 2 Materials and methods

### 2.1 Study data

The data were obtained from the GBD 2019 study. The Global Burden of Diseases, Injuries and Risk Factors Study provided a systematic assessment of health loss for 369 diseases and injuries, the age-and sex-specific mortality for 286 causes, and 87 risk factors in 204 countries and territories from 1990 to 2019, with a wide array of standardized analytical procedures, including data screenings, data adjustments to improve the quality and the comparability of the study (1). The data on CKD burden attributable to high sodium intake from 1990 to 2019 were took through the Global Health Data Exchange GBD Outcomes Tool,^1^ including the number of deaths, DALYs, age-standardized death rates (ASDRs) and age-standardized DALYs rates. In addition, we incorporated the estimates of the CKD disease burden attributable to high sodium intake in separate provinces in China from 1990 to 2019, as compiled by the National Center for Food Safety Risk Assessment of China (CFSA), and the database was processed primarily based on raw data from original sources, including the Disease Surveillance Points system in China, the cause-of-death reporting system collected by the Chinese Center for Disease Control and Prevention, medical certification of causes of death for Macao and Hong Kong, and nationwide surveys. To guarantee the reliability of the study, the data underwent a systematic processing procedure utilizing the DisMod-MR 2.1 model (Bayesian meta-regression model) (20).

The study covered 31 provinces, municipalities, and autonomous regions of China, as well as the Hong Kong and Macao Special Administrative Regions. Provinces were categorized employing the Sociodemographic Index (SDI) to assess the association between the SDI and the disease burden. SDI is a composite measure comprising three components: the total fertility rate for individuals under the age of 25 years, the average educational attainment of persons over the age of 15 years, and the average per capita lagged-distributed income. The SDI is distributed on a scale of 0 to 1, representing the lowest to highest level of development of the theoretical possession of health outcomes in a region (1, 21). Specifically, China’s SDI is 0.71, with a distribution across provinces ranging from 0.4 to 0.9. This distribution can be categorized into four classes: High SDI regions (Hong Kong, Macau, Beijing, Shanghai, and Taiwan); High-middle SDI regions (Fujian, Guangdong, Hebei, Heilongjiang, Inner Mongolia, Jiangsu, Jilin, Liaoning, Shandong, Tianjin, and Zhejiang); Low-middle SDI regions (Gansu, Guizhou, and Tibet) and Middle SDI regions (other regions).

The database is publicly open access and does not contain personal information, and there is no need to conduct a Research Ethics Committee for approval.

### 2.2 Definitions of CKD and high sodium intake

GBD defines CKD based on a single measurement of eGFR and ACR. Due to the limitations of epidemiological surveys, GBD does not synthesize other markers of kidney injury (7). ICD-9 mapped to CKD include 250.4, 403–404.9, 581–583.9, 585–585.9, 589–589.9, and 753–753.3; ICD-10 codes include D63.1, E10.2, E11.2, E12.2, E13.2, E14.2, and I12-I13.9, N02-N08.8, N15.0, N18-N18.9, and Q61-Q62.8 (22). In the study, an average 24-h urinary sodium excretion (in grams per day) greater than 3 g (95%UI:1 g,5 g) was defined as an indicator of exposure to high sodium intake (23).

### 2.3 Estimation of high sodium intake-attributed CKD burden

The details of estimates of attributable disease burden presented in the GBD2019 report have been described in previous articles (1, 7, 21, 23). Totally, the attributable burden was evaluated in accordance with a comparative risk assessment framework. CKD burden attributable to high sodium intake was initially identified based on the evidence provided by systematic reviews and meta-analyses across the globe. Based on those published articles, household surveys, census, administrative data, the exposure level and distribution of high sodium intake by age, sex, geographic area and year were estimated using spatio-temporal Gaussian process regression or DisMod-MR 2.1; then the theoretical minimum risk exposure level (TMREL) was determined as the exposure level associated with the lowest risk; Population attributable fractions (PAFs) by age, sex, geographic area and year were calculated according to the risk function, exposure level and TMREL. The standard GBD PAF equation is defined as follows:

$$PAF_{\mathrm{asgt}}=\frac{\sum_{x=l}^{u}RR_{asg}\left(x\right)P_{\mathrm{asgt}}\left(x\right)-RR_{asg}\left(\mathrm{TMRE}L_{as}\right)}{\sum_{x=l}^{u}RR_{as}\left(x\right)P_{\mathrm{asgt}}\left(x\right)}$$

Where PAFasgt was the PAF for CKD burden attributable to high sodium intake for age group a, sex s, geographic area g, and year t. RRast (x) was the relative risks between exposure level x (from l to u) of high sodium intake and CKD for age group a, sex s, and year t; and Pasgt (x) was the proportion of the population exposed to high sodium intake at the level x for age group a, sex s, geographic area g, and year t. TMRELas is the TMREL for age group a, and sex s.

Furthermore, potential mediating effect must be taken into account when assessing the effect. The attributable burden of CKD disease resulting from high sodium intake was calculated by multiplying the corresponding PAF for each age, sex, region and year by the total number of deaths from CKD and the number of years lived with disability.

### 2.4 Statistical analyses

The burden of CKD disease attributable to high sodium intake was quantified using a number of metrics, including the number of deaths, DALYs, age-standardized mortality rates (ASMR) and age-standardized DALYs rates (ASDR). The aforementioned data are represented by numerical values accompanied by 95% uncertainty intervals (UIs). 95% UI (uncertainty intervals) were generated for each metric using the 25th and 975th ordered 1000th plotted values of the posterior distribution. To describe the long-term trend in CKD disease burden attributable to high sodium intake from 1990 to 2019, the estimated annual percentage change (EAPC), a widely used metric for responding to trends in age-standardized rates (ASR), was calculated. ASR was fitted using the regression model ln (ASR) = *α* + *β*x + *ε*, where x denotes the calendar year (assuming natural logarithmic linear change in the ASR). EAPC and its 95% confidence interval (95% CI) were then derived on a logarithmic scale based on the model 100 × [exp(β)-1]. A positive lower 95% CI of the EAPC estimate indicates an upward trend in the ASR (ASMR, ASDR), while a negative upper 95% CI indicates a downward trend. However, the ASR is considered stable if the 95% CI of the EAPC estimate contains zero. Concurrently, we examined the correlation between EAPC and ASR and SDI through a smoothed spline model and Spearman’s test, and investigated the factors influencing the changes in the burden of disease in CKD attributable to high sodium intake. All statistical analyses were conducted using R software (version 4.1.2), and *p* < 0.05 was considered statistically significant.

## 3 Results

### 3.1 Deaths and ASMR of CKD attributable to high sodium intake

Over the past three decades, the global number of deaths from CKD attributed to high sodium intake has increased from 43,525.94 in 1990 to 95,876.08 in 2019, representing an obvious increase of 120.27%. Also, the ASMR has concurrently increased from 1.18 (95% UI: 0.32, 2.60) per 100,000 population in 1990 to 1.21 (95% UI: 0.25, 2.96) in 2019, with an estimated average annual increase of 0.22% (95% CI: 0.16, 0.28%) (Table 1).

**Table 1:** Deaths and ASMR of chronic kidney disease attributable-high sodium intake in 1990 and 2019 and the temporal trends from 1990–2019.

Characteristics	1990		2019			1990–2019
	Deaths cases, No. (95% UI)	ASMR per 100,000 No. (95% UI)	Deaths cases, No. (95% UI)	ASMR per 100,000 No. (95% UI)	PAFs % (95% UI)	EAPC (%) in ASMR No. (95% CI)
Global	43,525.94 (12,639.18 to 93,828.26)	1.18 (0.32 to 2.60)	95,876.08 (19,975.39 to 230,306.89)	1.21 (0.25 to 2.93)	4.54 (0.51 to 12.72)	0.22 (0.16 to 0.28)
China	14,911.09 (6,753.38 to 25,100.60)	1.90 (0.80 to 3.35)	29,665.73 (11,330.58 to 54,546.97)	1.56 (0.56 to 2.96)	0.14 (0.05 to 0.25)	-0.31 (−0.46 to-0.17)
Gender (China)						
Male	8,587.01 (4,155.37 to 14,025.44)	2.44 (1.08 to 4.21)	18,208.14 (7,631.05 to 31,626.31)	2.16 (0.81 to 3.89)	0.15 (0.06 to 0.27)	0.07 (−0.10 to 0.25)
Female	6,324.08 (2,445.69 to 11,495.34)	1.55 (0.55 to 2.91)	11,457.59 (3,163.15 to 23,374.18)	1.13 (0.30 to 2.34)	0.12 (0.03 to 0.24)	-0.85 (−0.98 to-0.72)
Socio-demographic Index (SDI)						
High SDI	550.95 (245.73 to 923.28)	2.13 (0.86 to 3.71)	1008.86 (341.46 to 1947.49)	1.20 (0.39 to 2.34)	0.14 (0.05 to 0.26)	−2.09 (−2.21 to-1.96)
High-middle SDI	5,825.85 (2,591.90 to 10,048.16)	1.85 (0.75 to 3.32)	10,790.29 (4,000.36 to 20,061.62)	1.29 (0.44 to 2.45)	0.14 (0.05 to 0.26)	−1.03 (−1.18 to-0.88)
Middle SDI	7,283.57 (3,405.25 to 13,462.10)	2.05 (0.80 to 3.72)	16,434.45 (5,899.14 to 30,855.96)	1.96 (0.67 to 3.44)	0.14 (0.05 to 0.25)	0.30 (0.12 to 0.48)
Low-middle SDI	763.14 (318.24 to 1364.62)	2.89 (1.21 to 5.23)	1,432.12 (451.19 to 2,811.58)	2.51 (0.91 to 4.77)	0.13 (0.05 to 0.24)	−0.36 (−0.46 to-0.26)

ASMR = age-standardized mortality rate; PAF = population attributable fraction; EAPC = estimated annual percentage change.

The number of CKD deaths attributable to high sodium intake increased from 14,911.09 in 1990 to 29,665.73 in 2019, representing a 98.95% increase over the period. However, the ASMR showed a declining trajectory, from 1.90 (95% UI: 0.80, 3.35) per 100,000 population in 1990 to 1.56 (95% UI: 0.56, 2.96) per 100,000 population in 2019, representing an estimated average decrease of 0.31% (95% CI: −0.46, −0.17%) per year. A similar characterization was observed in the data across different gender. ASMR in both males and females declined in 2019 compared to 1990, but the number of deaths definitely increased. ASMR in males decreased from 2.44 (95% UI: 1.08, 4.21) per 100,000 population in 1990 to 2.16 (95% UI: 0.81, 3.89) in 2019, obviously a stable trend, with EAPC of 0.07% (95% CI: −0.10, 025%); ASMR in females decreased from 1.55 (95% UI: 0.55, 2.91) per 100,000 population in 1990 to 1.13 (95% UI: 0.30, 2.34) in 2019, with a downward trend between 1990 and 2019 and an estimated annual percentage change (EAPC) of−0.85% (95% CI: −0.98, −0.72%). It is noteworthy that both deaths and ASMR were consistently higher in males than in females, and the downward trend was more pronounced in females (Table 1).

At the SDI level, the number of deaths in middle SDI areas was at the highest in both 1990 and 2019, and the lowest in high SDI areas. After adjusting for age-structure differences, the ASMR was found to be the highest in the low-middle SDI regions in 1990, followed by the high SDI regions, and the lowest in the high-middle SDI regions. In 2019, the ASMR in low-middle SDI regions remained the highest, while the ASMR in high SDI regions was the lowest. Interestingly, the middle SDI region exhibited an increasing trend in ASMR, with EAPC of 0.30% (95% CI: 0.12%, 0.48) between the 2 years of 1990 and 2019. The other three SDI regions showed a declining trend in ASMR and the high SDI region exhibiting the most notable decrease with an average annual decline of 2.09% (95% CI: −2.21, −1.96%) (Table 1).

**Figure 1:**
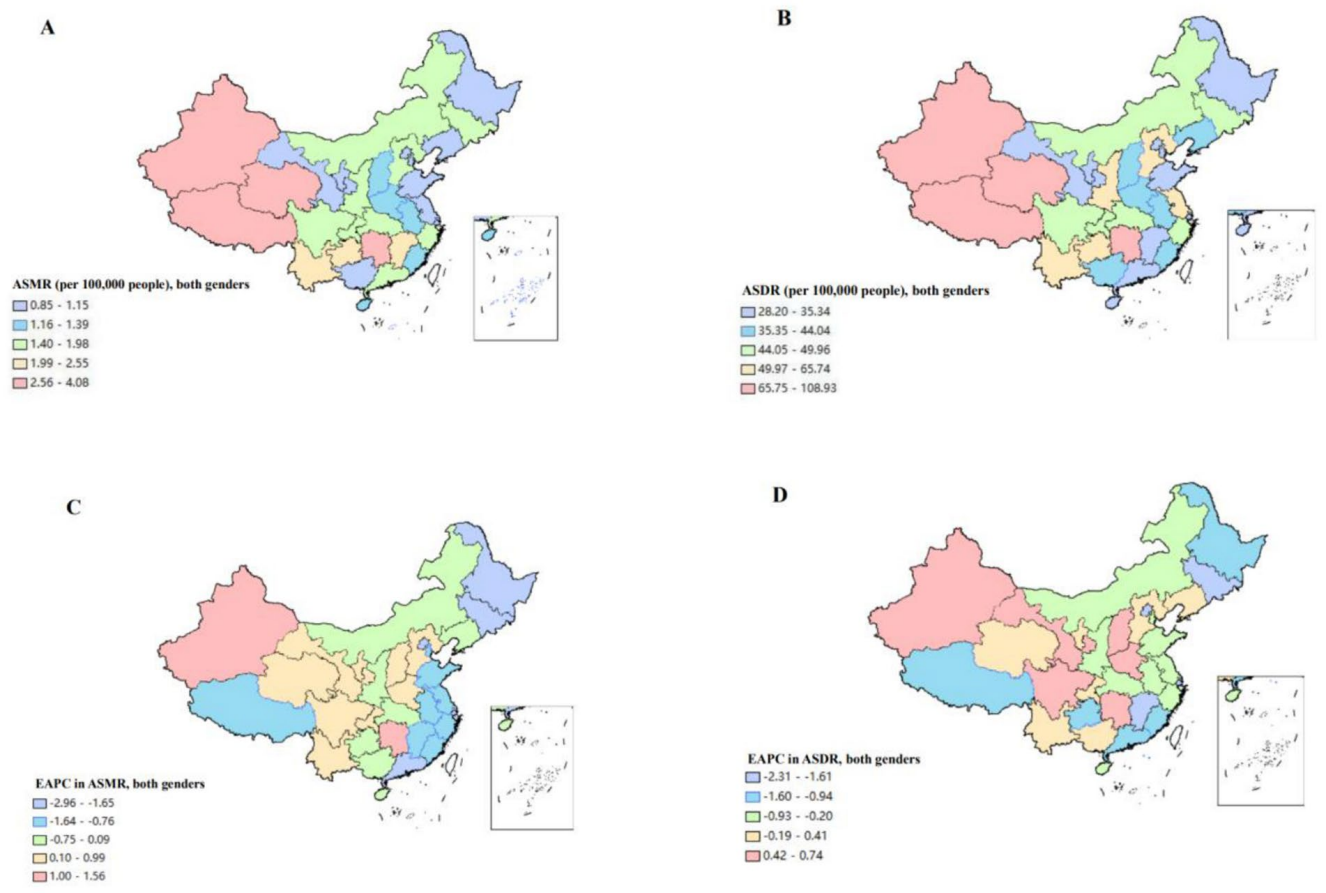
The disease burden of chronic kidney diseases attributable to high sodium intake for both genders combined in China. **(A)** The spatial distribution of chronic kidney diseases ASMR attributable to high sodium intake in 2019. **(B)** The spatial distribution of chronic kidney diseases ASDR attributable to high sodium intake in 2019. **(C)** The EAPC in chronic kidney diseases ASMR attributable to high sodium intake from 1990 to 2019. **(D)** The EAPC in chronic kidney diseases ASDR attributable to high sodium intake from 1990 to 2019. ASMR, age-standardized mortality rate; DALYs, disability-adjusted life years; ASDR, age-standardized DALYS rate; EAPC, estimated annual percentage.

At the provincial level, the number of deaths of CKD attributable to high sodium intake in 1990 and 2019 concentrated in Hunan, Sichuan, Henan, and Hebei, collectively accounting for approximately 30% of the total CKD deaths attributable to high sodium intake in China. Hunan had the highest number of deaths, at 2,922.05, in 2019, followed by Sichuan, which had 2,122.45 deaths in the same year. However, Macao and Ningxia had lower number of deaths. The proportion of CKD deaths attributable to high sodium intake in each province in 2019 ranged from 0.07 to 0.19%. Notably, Zhejiang, Tibet, and Anhui exhibited relatively elevated proportions, at 0.19, 0.18, and 0.17%, respectively. In terms of ASMR, Tibet, Jiangxi, and Jilin were in the top three in 1990, with ASMRs of 4.88 (95% UI: 2.30, 8.30), 4.04 (95% UI: 2.05, 6.51), and 2. 95 (95% UI: 1.38, 4.84) per 100,000 population, respectively. In 2019, Tibet had ASMR of 4.08 (95% UI: 1.78, 7.10) in pole position, followed by Hunan with ASMR of 3.10 (95% UI: 0.98, 6.01), with Qinghai ranking third with ASMR of 3.08 (95% UI: 1.28, 5.40) per 100,000 population. In contrast, the three provinces that had lower ASMR in 1990 were Gansu, Ningxia and Tianjin, with 0.92 (95% UI: 0.13, 2.27), 1.15 (95% UI: 0.27, 2.47) and 1.20 (95% UI: 0.40, 2.29), respectively. In 2019, the rankings shifted to Tianjin, Heilongjiang, and Shanghai, with ASMRs of 0.85 (95% UI: 0.23, 1.74), 0.95 (95% UI: 0.26, 1.97), and 0.98 (95% UI: 0.31, 1.94). ASMR of CKD attributable to high sodium intake in 2019, as illustrated in the heat map, revealed that the values were predominantly higher in the inland areas of western and southern China, with relatively lower values observed in the coastal areas. Moreover, within the period of 1990 and 2019, 11 provinces demonstrated an upward trend in ASMR. Among these regions, Xinjiang and Hunan exhibited the most pronounced increase with an EACP of 1.56% (95% CI: 1.35, 1.78%). The other 19 provinces exhibited a declining trend in ASMR, while Beijing and Shanghai demonstrated the most obvious reduction with the EAPC of −2.96% (95% CI: −3.22, − 2.70%) and −2.15 (95% CI: −2.31, −2.00), respectively (Supplementary Table 1; Figures 1A,C).

### 3.2 DALYs and ASDR of CKD attributable to high sodium intake

Over the past three decades, the global DALYs for CKD attributable to high sodium intake have increased from 1,318,805.68 (95% UI: 429,236.90, 2,753,297.76) in 1990 to 2,590,152.43 (95% UI: 636,970.05, 5,865,231.50) in 2019, representing a 96.4% increase. Although a slight decrease was observed in 2019, compared to 1990, ASDR still exhibited an upward trend between 1990 and 2019, with an estimated average annual increase of 0.10% (95% CI: 0.04, 0.16%) (Table 2).

**Table 2:** DALYs and ASDR of chronic kidney disease attributable-high sodium intake in 1990 and 2019 and the temporal trends from 1990–2019.

Characteristics	1990		2019		1990–2019	
	DALYs, No. (95% UI)	ASDR per 100,000 No. (95% UI)	DALYs, No. (95% UI)	ASDR per 100,000 No. (95% UI)	PAFs % (95% UI)	EAPC (%) in ASDR No. (95% CI)
Global	1,318,805.68 (4,292,36.90 to 27,53,297.76)	32.19 (10.18 to 67.66)	2,590,152.43 (636,970.05 to 5,865,231.50)	31.43 (7.64 to 71.63)	3.80 (0.48 to 10.21)	0.10 (0.04 to 0.16)
China	528,326.02 (264,395.53 to 859,685.81)	57.38 (27.40 to 95.15)	922,143.09 (407,985.79 to 1,570,598.14)	45.41 (19.55 to 78.19)	0.15 (0.06 to 0.24)	−0.33 (−0.48 to-0.18)
Gender (China)						
Male	304,900.14 (157,335.61 to 482,520.99)	40.57 (13.79 to 82.87)	559,686.55 (267,881.00 to 922,408.57)	57.48 (26.60 to 96.62)	0.16 (0.08 to 0.27)	−0.03 (−0.19 to 0.13)
Female	223,425.89 (95,887.46 to 384,364.33)	25.27 (6.95 to 56.47)	362,456.53 (125,798.77 to 671,143.37)	34.77 (12.00 to 65.13)	0.12 (0.04 to 0.23)	−0.74 (−0.89 to-0.59)
Socio-demographic Index (SDI)						
High SDI	17,844.17 (8,767.32 to 28612.35235)	59.25 (27.72 to 97.36)	30,204.81 (12,751.23 to 52,974.16)	35.12 (14.45 to 62.27)	0.15 (0.06 to 0.25)	−1.58 (−1.74 to-1.42)
High-middle SDI	206,775.89 (101,144.08 to 341,059.54)	55.52 (26.05 to 93.15)	362,404.05 (158,534.19 to 624,652.66)	39.97 (16.99 to 69.50)	0.15 (0.06 to 0.25)	−0.68 (−0.86 to-0.51)
Middle SDI	276,282.70 (131,901.20 to 461,006.40)	61.81 (27.53 to 106.20)	487,948.10 (205,733.60 to 851,131.50)	53.82 (22.25 to 95.05)	0.14 (0.06 to 0.25)	0.02 (−0.14 to 0.17)
Low-middle SDI	27,423.24 (12,186.90 to 47,371.41)	88.52 (41.30 to 150.40)	41,586.11 (15,510.93 to 76,292.00)	66.85 (28.66 to 116.48)	0.14 (0.06 to 0.23)	−0.96 (−1.31 to-0.61)

DALYs = disability-adjusted life years; ASDR = age-standardized DALYs rate; PAF = population attributable fraction; EAPC = estimated annual percentage change.

The number of DALYs of CKD attributed to high sodium intake increased from 528,326.02 (95% UI: 264,395.53, 859,685.81) in 1990 to 922,143.09 (95% UI: 407,985.79, 1,570,598.14) in 2019, which represented an increase of 74.54%. ASDR decreased from 57.38 (95% UI: 27.40, 95.15) per 100,000 person-years in 1990 to 45.41 (95% UI: 19.55, 78.19) per 100,000 person-years in 2019, with an estimated average annual decline of 0.33% (95% CI: −0.48, −0.18%). Males had higher DALYs and ASDR than females, which was similar to deaths and ASMR. And, there was a more pronounced downward trend in ASMR for females, with an average annual decrease of 0.74% (95% CI: −0.89, −0.59%) between 1990 and 2019. However, the trend was stable for males with an EPAC of −0.03% (95% CI: −0.19, 0.13%) (Table 2).

At the SDI level, it is totally similar for deaths and ASMR. Low-middle SDI regions had higher ASDR, while middle SDI regions had the highest DALYs, and the trend was stable in middle SDI regions compared to the other three regions with decreasing trends in ASDR (Table 2).

At the provincial level, the DALYs for CKD attributed to high sodium intake in 1990 were concentrated in Sichuan, Hunan, Shandong, and Jiangsu, with Sichuan ranking first at 41,539.75. In 2019, the primary concentrations were observed in Sichuan, Hunan, Shandong, Hebei, and Henan, with Hunan ranking first at 79,096.73. Macao and Ningxia represented the lowest levels of DALYs in both 1990 and 2019. This is consistent with the profile of deaths. Additionally, proportion of DALYs in CKD attributed to high sodium intake expressed as a percentage of the total DALYs separated by province in 2019 ranged from 0.08 to 0.20%. Specifically, Zhejiang, Tibet, and Anhui exhibited the highest proportions of DALYs, at 0.20, 0.18, and 0.18%, respectively.

In terms of ASDR, Tibet, Jiangsu, and Guizhou had the highest rate in 1990, with ASDR of 148.9 (95% UI: 76.52, 242.1), 116.91 (95% UI: 64.11, 182.93) and 89.12 (95% UI: 42.08, 147.75) per 100,000 person-years, respectively. In 1990, the top three were held by Tibet, Jiangsu, and Guizhou, with ASDR of 148.9 (95% UI: 76.52, 242.1), 116.91 (95% UI: 64.11, 182.93) and 89.12 (95% UI: 42.21, 147.75), respectively. In 2019, the three provinces which had the highest ASDR were Tibet, Qinghai, and Xinjiang, with rate of 108.93 (95% UI: 54.32, 176.25), 81.25 (95% UI: 38.28, 134.20), and 80.06 (95% UI: 38.4, 135.08), respectively. In contrast, Gansu, Tianjin and Ningxia had lower ASDR in 1990; whereas in 2019, provinces with lower ASDR shifted to Gansu, Tianjin and Shanghai. As observed in the heat map, ASDR exhibited a pattern similar to that of ASMR, which was predominantly concentrated in the inland regions of western and southern China. The coastal areas exhibited a relatively lower ASDR. During the period from 1990 to 2019, 10 provinces exhibited an increasing trend in ASDR. Among these, Xinjiang and Hunan expressed the most pronounced increase, with an EACP of 0.74% (95% CI: 0.58, 0.90%) and 0.72% (95% CI: 0.59, 0.86%), respectively; 20 provinces exhibited a declining trend in ASDR, among which Beijing and Shanghai had a more pronounced decline, with EAPC of −2.31% (95% CI: −2.50, −2.12%) and −1.79 (95%CI: −2.00, −1.58); Other three provinces demonstrated a stable trend (Supplementary Table 2; Figures 1B,D).

### 3.3 Deaths and ASMR of CKD attributable to high sodium intake by age and gender

**Figure 2:**
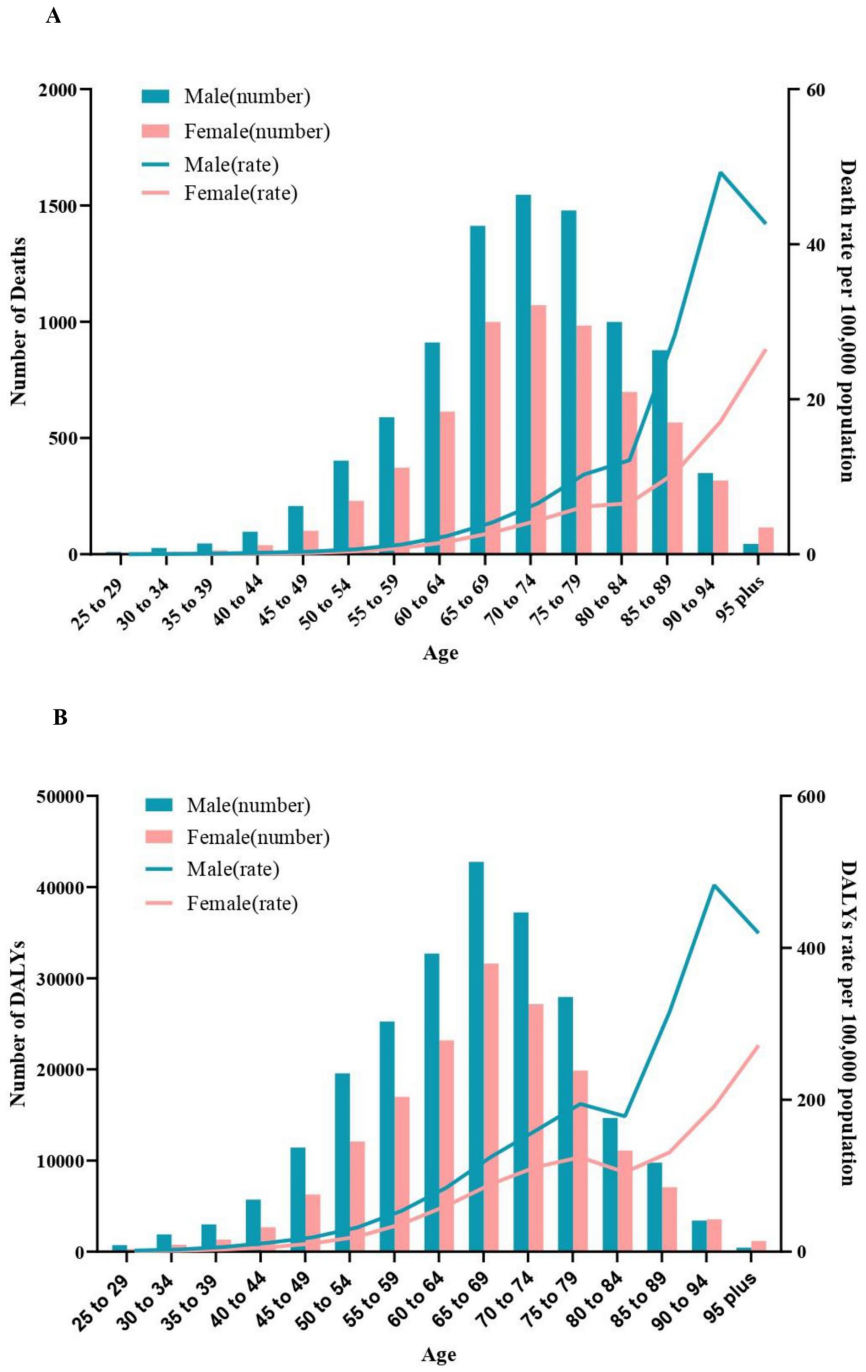
Age-specific numbers and rates of chronic kidney diseases deaths and DALYS attributable to high sodium intake by sex, in 2019. **(A)** Deaths. **(B)** DALYs. DALYS, disability-adjusted life years.

In 2019, the number of deaths for CKD burden attributed to high sodium intake increased until the age of 70 years, peaked in the age group of 70–74 years, and then decreased. Deaths among males exceeded that of females in all age groups except for those aged 95 and above. Both male and female age-specific mortality rates demonstrate an increasing trend with age, with gender differences becoming progressively more pronounced (males exhibit a higher mortality rate than females), and both exhibited a sharp increase at 80–84. Compared to females, males had a reduction in mortality rates following the age of 90 years (Figure 2A).

**Figure 3:**
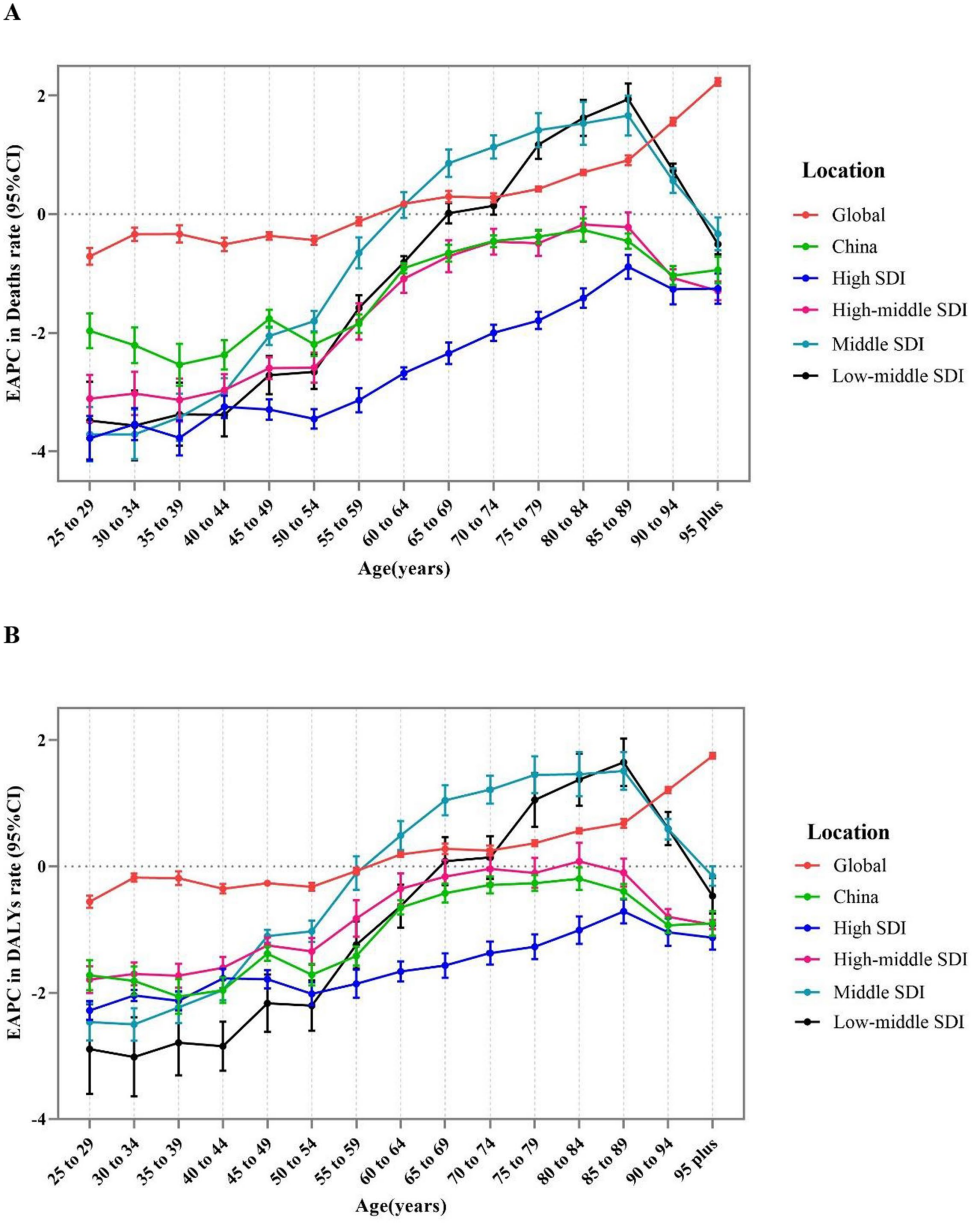
The age distribution of the trends in chronic kidney diseases-related mortality rate and DALYS rate attributable to high sodium intake from 1990 to 2019 by location. **(A)** EAPC in mortality rate. **(B)** EAPC in DALYS rate. DALYs, disability-adjusted life years; EAPC, estimated annual percentage change.

At the SDI level, the EAPC for age-specific mortality rate in four SDI regions increased with age, followed by declining after the age of 90. Particularly, the EAPC for age-specific mortality in the high SDI and high-middle SDI regions was less than zero, indicating that age-specific mortality from CKD due to high sodium intake presented an overall decreasing trend in these two regions from 1990 to 2019. However, the decreasing trend gradually became light with increasing age. The EAPC was positive for ages 65–90 in middle SDI and Low-middle regions, while it was negative for all other age groups and reached the maximum in 85–89 age group. Moreover, the most obvious decline in age-specific mortality across the four SDI regions was detected among individuals aged 30–39 (Figure 3A).

### 3.4 DALYs and ASDR of CKD attributable to high sodium intake by age and gender

In 2019, DALYs for CKD attributed to high sodium intake in males and females peaked in the age of 65–69 years, which was slightly earlier than the number of deaths. Males had higher DALYs than females in all age groups except age ≥ 90. Age-specific DALYs rates for males and females exhibited a similar trend, although there was a brief decline observed among those aged 75–85 (Figure 2B).

At the SDI level, changes in age-specific DALY rates exhibited a similar profile compared to the mortality rate (Figure 3B).

### 3.5 The association between ASMR and ASDR of CKD attributable to high sodium intake and SDI

**Figure 4:**
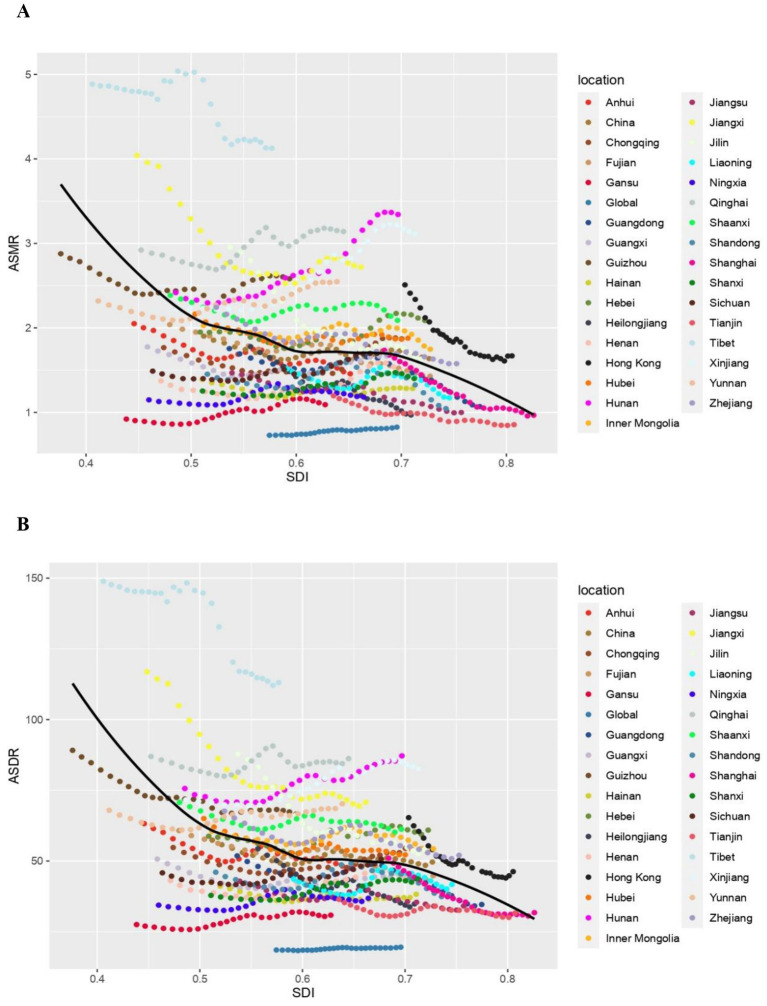
The chronic kidney diseases-related ASMR and ASDR attributable to high sodium intake across 33 provinces Burden of Disease regions by socio-demographic index for both sexes combined, 1990–2019. For each region, points right depict estimates from each year from 1990 to 2019. **(A)** The correlation between chronic kidney diseases-related ASMR attributable to high sodium intake and socio-demographic index. **(B)** The correlation between chronic kidney diseases-related ASDR attributable to high sodium intake and socio-demographic index. ASMR, age-standardized mortality rate; DALY, disability adjusted life year; ASDR, age-standardized DALY rate.

The pattern of ASMR and ASDR in CKD attributable to high sodium intake related to SDI exhibited a striking similarity from 1990 to 2019. Totally, ASMR and ASDR declined rapidly with increasing SDI when SDI was less than 0.5. Between 0.5 and 0.7, the change with the rise of SDI was relatively smooth, showing a slow decline. When SDI was greater than 0.7, the decline rate accelerated once more. Specifically, as for each province, the ASMR and ASDR of Hunan and Xinjiang demonstrated a notable increase with the rise of SDI. ASMR and ASDR of Tibet, Jiangxi, Hong Kong, Jilin, Qinghai, and Shaanxi remained stable, but they were still higher than the expected level, with the highest values in Tibet. In contrast, the remaining provinces exhibited the level lower or close to the anticipated level, as is notable for Guizhou and Shaanxi. It is noteworthy that the decreasing trend of ASMR and ASDR in Guizhou and Henan was less pronounced than anticipated as the SDI increased. However, in the subsequent period, these rates demonstrated a more pronounced increase than expected (Figures 4A,B).

**Figure 5:**
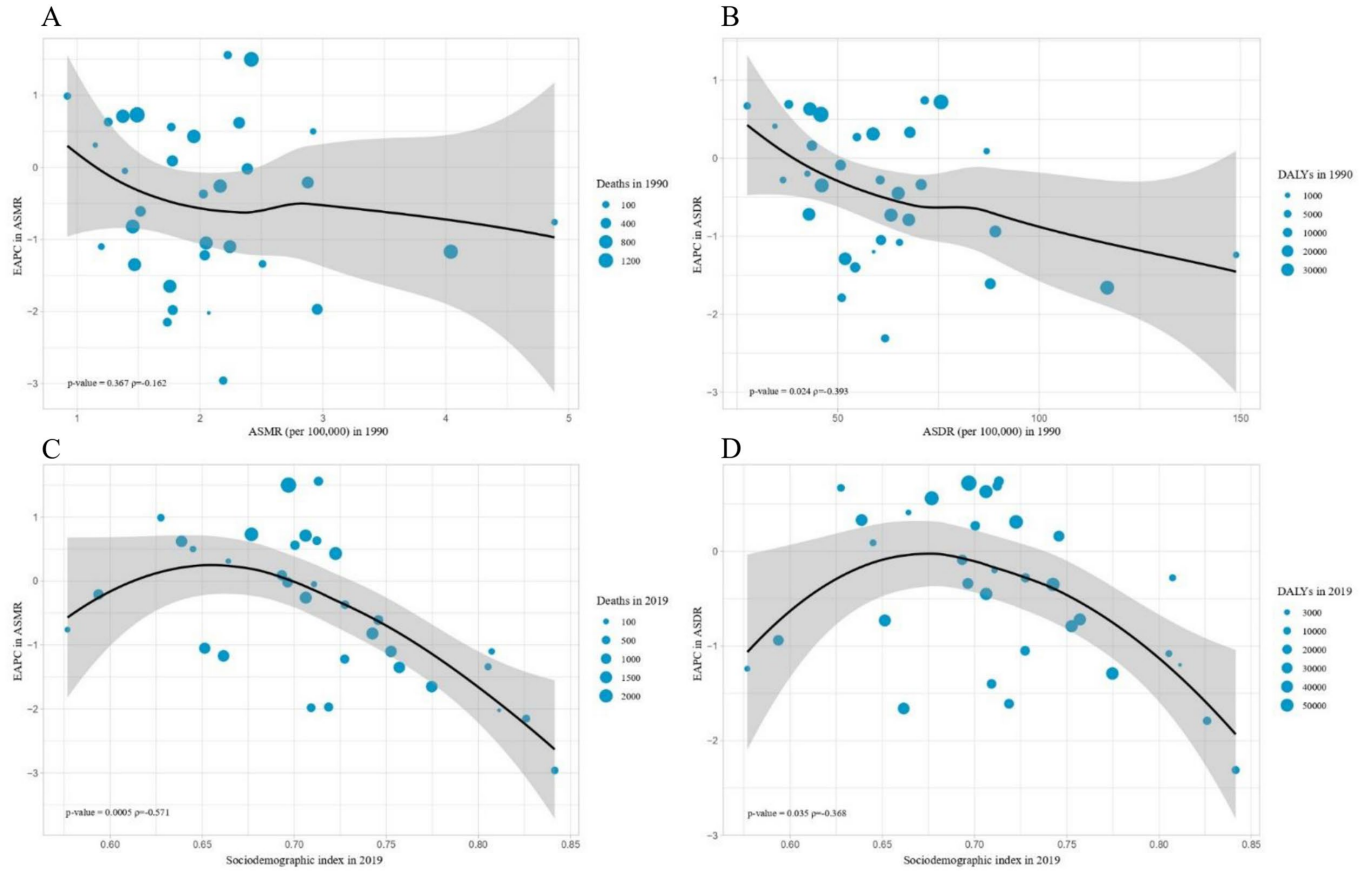
The influential factors for EAPC. **(A)** The correlation between EAPC in ASMR and ASMR in 1990. **(B)** The correlation between EAPC in ASDR and ASDR in 1990. **(C)** The correlation between EAPC in ASMR and sociodemographic index in 2019. **(D)** The correlation between EAPC in ASDR and sociodemographic index in 2019. The circles represent countries and the size of circle is increased with the number of chronic kidney diseases-related deaths and DALYS attributable to high sodium intake. The p indices and *p* values presented in **(C,D)** were derived from Pearson correlation analysis. ASMR, age-standardized mortality rate; DALYS, disability-adjusted life years; ASDR, age-standardized DALYS rate; EAPC, estimated annual percentage change.

Furthermore, a strong negative correlation was detected between the EAPC of ASDR and the ASDR of CKD in 1990 (*ρ* = −0.393, *p* = 0.024). However, no significant correlation was detected between the EAPC of ASMR and the ASMR of CKD in 1990 (*ρ* = −0.162, *p* = 0.367) (Figures 5A,B). Additionally, a significant negative correlation was detected between the EAPC in ASRs of deaths and DALYs and the SDI in 2019(ASMR: *ρ* = −0.571, *p* < 0.001; ASDR: *ρ* = −0.368, *p* = 0.035) (Figures 5C,D).

## 4 Discussion

As an essential nutrient for maintaining basic physiological functions, sodium is primarily obtained from food-added salt (19). High sodium intake is a crucial risk factor for the development and progression of CKD. The latest spatiotemporal patterns of CKD burden attributable to high sodium intake still remain unclear in China because of variations among regions, as well as complex associations with sociodemographic factors. We aimed to evaluate the level and trends of the CKD burden associated with high sodium intake according to sex, age, socio-demographic index (SDI), regions in China from 1990 to 2019.

The CKD burden (Deaths, DALYs) attributable to high sodium intake in China in 2019 is approximately twice as high as it was in 1990, and China’s proportion is higher than that of globality. In China, approximately 70% of sodium intake is derived from food-added salt, with the remainder from seasonings, such as soy sauce and chicken essence (24). Despite the implementation of the national salt reduction policy, the salt intake of Chinese residents remains at a relatively high level (25). Proliferation of convenient fast food, take-out, and restaurants has become a dominant feature of the food landscape, offering a vast array of flavor-oriented foods that present a formidable challenge to reduce salt intake. A cross-sectional survey revealed that, in comparison with homemade dishes, restaurant dishes contained a higher level of sodium in China (24). These dishes encompass a multitude of sodium sources, extending beyond regular table salt to encompass flavor enhancers and seasonings (26). The complexity renders salt-specific sodium reduction strategies less efficacious. In light of the persistently elevated sodium intake in China and the rising influence of the “convenience dining” culture, it is imperative to ascertain the feature of current CKD disease burden attributable to high sodium intake and promote effective prevention and control strategies.

Our findings indicate that CKD burden attributable to high sodium intake is higher in males than in females across the majority of age groups in China. However, this trend reverses in individuals above the age of 90, potentially due to a higher average life expectancy in females compared to males. These observations align with previous studies, suggesting that males may be more susceptible to the adverse effects of high sodium intake (22, 27). This discrepancy could, in part, be attributed to the inherent differences in the internal milieu between males and females. The body ingests sodium and subsequently excretes it in the urine, with intake and excretion reaching a steady state within approximately 3 days (28). Interestingly, on the first day, the females excreted a greater quantity of sodium compared to the males, which may have been attributed to elevated levels of urinary endothelin-1 (28). This indicates that females are capable of responding more rapidly to a high sodium environment. Similarly, the magnitude of urinary sodium excretion was found to be greater in females in high sodium environments (29). Female distal convoluted tubules demonstrate enhanced structural remodeling capacity to elongate in response to loop diuretics, indicating the existence of distinctive physiological mechanisms enabling females to cope with elevated sodium levels (30). Presumable evidence indicates that the progression of CKD is influenced by estrogen in females (31). Additionally, the difference of lifestyle between males and females also accounts for the onset and progression of CKD. In China, men tend to engage in more social activities and dine out for business purposes. Restaurant meals typically contain higher levels of sodium compared to home-cooked dishes (24). Also, men are more prone to adopt unhealthy lifestyle habits, such as tobacco use and excessive alcohol consumption, which collectively exacerbate the health risks associated with high sodium intake. Consequently, it is essential to enhance health education initiatives, as well to establish a lower recommended daily sodium intake for males’ gender.

CKD burden attributable to high sodium intake is more serious in the older adult. Seniors predispose to hypertension, cardiovascular disease (CVD) and diabetes, thereby increasing CKD risk. Over 10% of Chinese residents were over the age of 60, and this trend is on the rise in 2019 (20). Since altered vascular elasticity and decreased renal filtration rates are observed as the age advances, the impaired function renders the elder less able to utilize excessive sodium intake. Moreover, researches indicated that many risk factors of CVD exacerbate the development of CKD, and CKD patients have a higher risk of developing increased cardiovascular morbidity and mortality. Interestingly, compelling studies suggest that multiple traditional and novel risk factors in CKD patients predispose to CVD morbidity and mortality, strengthening the assumption that these two diseases are inherently interrelated with each other (32, 33). Common potential risk factors between CVD and CKD include age, hypertension, diabetes mellitus, dyslipidemia, tobacco use, high sodium intake and male gender (34). Despite ongoing efforts to reduce salt intake, the idea that seniors are more vulnerable to the adverse effect of high sodium consumption are still prevailing. This underscores the necessity for tailored policies and prevention strategies to enhance awareness about salt restriction among the older adult.

At the provincial level, CKD burden attributable to high sodium intake and its trends exhibit variations across provinces. Hunan has the highest number of deaths and DALYs of CKD attributed to due to high sodium intake in both 1990 and 2019. Both ASMR and ASDR show a notable upward trend throughout the observation period. Hunan cuisine is characterized by chili peppers used extensively, along with high intake of oil and salt and a preference for salt-cured meat products (35). The sodium intake of individuals in Hunan exceeds the recommended levels (36). It is reasonable that the high salt intake has contributed to the increased CKD burden in Hunan. This dietary habit is deeply rooted for a long time, resulting in a significant upward trend in its ASMR and ASDR. Moreover, as for ASMR and ASDR, the attributed CKD burden exhibits a North–South disparity with a greater magnitude in the southern region compared to the northern. This discrepancy could be attributed to the temperature patterns between the two regions. One study in Asian reported that short-term mortality due to heat stress, also heat exposure significantly affects the long-term survival and prognosis of CKD patients, with everlasting effect for years (37).

A similarly severe disease burden was detected in the western China. In the case of Tibet, this could be related to the relatively lower economic level, lack of health awareness, and inadequate health system. Suboptimal economic and health conditions impede Tibetans’ access to timely and efficacious treatment for ailments, such as CKD, thereby exacerbating the disease burden. Limited educational resource impedes the popularization of health awareness. Also, Tibet is situated at an altitude of approximately 4,500 meters above sea level. Prolonged exposure to high altitude and low-oxygen environment could partly stimulate sympathetic and parasympathetic nerve activity, resulting in an elevation of blood pressure. Prolonged exposure to hypoxia for Tibetans can lead to alterations in the elasticity and diameter of blood vessels, potentially impacting blood supply to the kidney (38). In view of Tibetan diet, high consumption of meat, dairy products, and grains exhibit high intake of fat and sodium that exceeds the recommended level (39). Traditional Tibetan food, salted ghee tea, contains as much as four times the recommended level of salt (40). Interestingly, Macau, a relatively lower burden of CKD attributable to high sodium intake, presents a striking contrast to Tibet. One study in Macao revealed that the sodium intake is obviously lower among university students (41). A well-developed healthcare system, effective hygiene measures, and a high level of health awareness serve to reduce the burden of disease (42). Therefore, in those regions characterized by high sodium intake, it is imperative to establish relative stringent guidelines and disseminate information regarding the adverse effects of excessive sodium intake, thereby enhancing public health awareness among residents.

The CKD burden attributed to high sodium intake also exhibits variations across different SDI regions. Although high SDI regions originally exhibited the highest ASMR and ASDR, the disease burden showed a precipitous decline over time. High SDI region, as the vanguard of national development, prioritizes the implementation of national policies. Superior healthcare system, as well as medical investment, education, and health awareness, decrease disease burden at a rapid pace. Medium SDI regions, as the only regions displaying an upward trend, continue to exhibit the high disease burden. Despite moderate level of health investment, these changes have not offset the negative impacts of changing habits, leading to an even worse situation. The CKD burden attributed to high sodium intake exhibited a rapid decline with decreasing SDI, as lower SDI regions are more susceptible to public policies. Therefore, CKD is largely preventable and treatable and deserves greater attention in health policy decision making, particularly those regions with low and middle SDI could be given priority to health investment.

In this study, utilizing summary data from a representative dataset, we conducted a comprehensive analysis of the temporal trends and spatial distribution disparities in the disease burden of CKD attributable to high sodium intake across provinces of China from 1990 to 2019. This study explored the epidemiological patterns of CKD in China and the overall impact of high sodium intake on health outcomes, making the evaluation more comprehensive and thoughtful as for variations of high sodium intake-related CKD burden separated by province in China. It is worth noting that, we employed 24-h urine collection to evaluate dietary sodium intake that was recognized for the reliability compared to dietary recall assessments. Nevertheless, some limitations still need to be considered in the study. First, despite the national vital registration system providing sufficient data on mortality and the incidence of CKD in China, data regarding non-fatal outcomes of CKD remain under-reported in remote and economically disadvantaged regions, primarily due to the incomplete disease registration system. Second, the participants include individuals aged over 25, while the details of individuals aged 0–24 are unavailable since the risk of health outcome was relatively minimal. Moreover, except for high sodium intake, the CKD risk is influenced as by excess alcohol consumption, as well as unhealthy dietary habits, which may confound the results.

In summary, our findings suggest that there are significant sexual and geographic variations in CKD burden attributable to high sodium intake. Nationally, the high sodium intake-related CKD burden continues to elevate, posing a major challenge to public health. Despite a downward trend of disease burden exists in China, the absolute number increased as much as twofold, particularly among males and seniors. In response to this, more attention could be given that middle SDI regions that are experiencing rising trends and low-middle SDI areas are susceptible to approaches for CKD prevention.

## Data Availability

The original contributions presented in the study are included in the article/Supplementary material, further inquiries can be directed to the corresponding authors.

## Footnotes

^1^ https://vizhub.healthdata.org/gbd-results/
