# Supplementary material for: The burden of chronic kidney disease attributable to high sodium intake: a longitudinal study in 1990–2019 in China
Source: Front Nutr. 2025 Jan 15;11:1531358. doi: 10.3389/fnut.2024.1531358 (PMC11783680; doi:10.3389/fnut.2024.1531358)
Supplement: Supplementary file 1 [file Table_1.DOCX]

***Supplementary Tables 1*.** Deaths and ASMR of chronic kidney disease attributable-high sodium intake in 1990 and 2019 and the temporal trends from 1990-2019

| **Characteristics** | **1990** | | **2021** | | | **1990-2019** |
| --- | --- | --- | --- | --- | --- | --- |
|  | **Deaths cases,**  **No. (95% UI)** | **ASMR per**  **100,000 No. (95% UI)** | **Deaths cases,**  **No. (95% UI)** | **ASMR per**  **100,000 No. (95% UI)** | **PAFs %**  **(95% UI)** | **EAPC (%) in**  **ASMR No. (95% CI)** |
| **Region** | | | | | | |
| Anhui | 759.16 (382.38 to 1215.12) | 2.05 (0.96 to 3.38) | 1242.62 (514.03 to 2228.30) | 1.39 (0.56 to 2.53) | 0.17 (0.07 to 0.29) | -1.05 (-1.20 to -0.89) |
| Beijing | 184.78 (93.89 to 292.60) | 2.19 (1.04 to 3.58) | 336.34 (132.00 to 614.20) | 1.06 (0.40 to 1.96) | 0.16 (0.06 to 0.28) | -2.96 (-3.22 to- 2.70) |
| Chongqing | 204.18 (80.38 to 360.44) | 1.77 (0.65 to 3.24) | 739.97 (243.90 to 1430.63) | 1.75 (0.57 to 3.43) | 0.14 (0.05 to 0.25) | 0.56 (0.21 to 0.91) |
| Fujian | 383.91 (169.75 to 666.07) | 2.04 (0.81 to 3.69) | 667.43 (235.19 to 1243.61) | 1.39 (0.45 to 2.64) | 0.14 (0.05 to 0.27) | -1.22 (-1.35 to -1.08) |
| Gansu | 120.34 (21.15 to 273.49) | 0.92 (0.13 to 2.27) | 325.41 (57.62 to 746.10) | 1.03 (0.17 to 2.51) | 0.07 (0.01 to 0.17) | 0.99 (0.77 to 1.21) |
| Guangdong | 752.2 (254.96 to 1430.43) | 1.75 (0.53 to 3.47) | 957.33 (199.90 to 2086.01) | 1.59 (0.32 to 3.53) | 0.10 (0.02 to 0.20) | -1.65 (-1.99 to -1.31) |
| Guangxi | 487.58 (147.93 to 936.03) | 1.78 (0.49 to 3.53) | 1314.29 (383.42 to 2670.91) | 1.04 (0.28 to 2.16) | 0.11 (0.03 to 0.22) | 0.09 (-0.17 to 0.35) |
| Guizhou | 572.97 (261.87 to 975.18) | 2.88 (1.21 to 5.10) | 1003.28 (343.75 to 1894.47) | 2.41 (0.78 to 4.70) | 0.14 (0.05 to 0.25) | -0.21 (-0.42 to -0.01) |
| Hainan | 59.84 (18.61 to 121.27) | 1.39 (0.39 to 2.89) | 131.92 (29.80to 277.02) | 1.24 (0.25 to 2.69) | 0.10 (0.02 to 0.20) | -0.05 (-0.21 to 0.11) |
| Hebei | 824.95 (380.91 to 1399.07) | 1.95 (0.82 to 3.40) | 1867.45 (723.41 to 3358.74) | 1.98 (0.69 to 3.69) | 0.15 (0.05 to 0.26) | 0.43 (0.21 to 0.65) |
| Heilongjiang | 349.76 (146.49 to 610.58) | 1.78 (0.64 to 3.39) | 557.38 (169.65 to 1103.25) | 0.95 (0.26 to 1.97) | 0.11 (0.03 to 0.23) | -1.98 (-2.16 to -1.81) |
| Henan | 796.98 (373.40 to 1339.64) | 1.37 (0.59 to 2.41) | 1627.88 (653.25 to 2999.32) | 1.35 (0.51 to 2.56) | 0.15 (0.06 to 0.27) | 0.71 (0.42 to 1.00) |
| Hong Kong | 134.58 (53.24 to 231.02) | 2.51 (0.91 to 4.46) | 251.17 (69.05 to 514.76) | 1.63 (0.48 to 3.27) | 0.13 (0.04 to 0.25) | -1.34 (-1.54 to -1.14) |
| Hubei | 782.39 (358.39 to 1317.08) | 2.16 (0.90 to 3.81) | 1499.25 (497.18 to 2910.12) | 1.82 (0.55 to 3.67) | 0.13 (0.04 to 0.25) | -0.26 (-0.41 to -0.12) |
| Hunan | 1028.11 (433.40 to 1809.77) | 2.42 (0.94 to 4.47) | 2922.05 (975.75 to 5513.37) | 3.10 (0.98 to 6.01) | 0.14 (0.04 to 0.25) | 1.50 (1.30 to 1.69) |
| Inner Mongolia | 245.41 (116.83 to 409.84) | 2.03 (0.86 to 3.54) | 541.39 (232.89 to 934.96) | 1.69 (0.65 to 3.05) | 0.16 (0.07 to 0.28) | -0.37 (-0.56 to -0.19) |
| Jiangsu | 739.18 (336.62 to 1238.38) | 1.47 (0.60 to 2.57) | 1312.75 (483.12 to 2510.57) | 0.99 (0.35 to 1.91) | 0.15 (0.05 to 0.26) | -1.35 (-1.50 to -1.20) |
| Jiangxi | 951.24 (509.37 to 1502.89) | 4.04 (2.05 to 6.51) | 1356.74 (548.27 to 2394.38) | 2.55 (0.96 to 4.64) | 0.16 (0.06 to 0.28) | -1.17 (-1.56 to -0.79) |
| Jilin | 443.02 (226.09 to 696.85) | 2.95 (1.38 to 4.84) | 649.14 (242.86 to 1183.64) | 1.57 (0.53 to 2.94) | 0.14 (0.05 to 0.26) | -1.97 (-2.22 to -1.71) |
| Liaoning | 409.66 (142.01 to 796.01) | 1.52 (0.46 to 3.09) | 876.35 (285.30 to 1727.26) | 1.15 (0.35 to 2.33) | 0.13 (0.04 to 0.24) | -0.61 (-0.89 to -0.33) |
| Macao | 5.61 (2.23 to 9.85) | 2.07 (0.81 to 3.65) | 10.56 (3.75 to 20.03) | 1.13 (0.39 to 2.19) | 0.15 (0.05 to 0.27) | -2.02 (-2.25 to -1.79) |
| Ningxia | 26.22 (7.43 to 53.56) | 1.15 (0.27 to 2.47) | 79.89 (23.07 to 160.47) | 1.14 (0.29 to 2.37) | 0.11 (0.03 to 0.22) | 0.31 (0.08 to 0.54) |
| Qinghai | 66.98 (33.62 to 108.89) | 2.92 (1.33 to 4.89) | 181.97 (82.20 to 308.76) | 3.08 (1.28 to 5.40) | 0.16 (0.07 to 0.28) | 0.50 (0.36 to 0.65) |
| Shaanxi | 503.23 (225.65 to 852.61) | 2.39 (0.99 to 4.14) | 988.68 (415.01 to 1734.98) | 1.97 (0.77 to 3.57) | 0.16 (0.06 to 0.28) | -0.02 (-0.39 to 0.02) |
| Shandong | 882.03 (410.07 to 1504.23) | 1.45 (0.62 to 2.59) | 1511.67 (606.29 to 2756.63) | 1.01 (0.39 to 1.87) | 0.16 (0.06 to 0.28) | -0.82 (-1.29 to -0.36) |
| Shanghai | 225.99 (96.37 to 389.80) | 1.73 (0.66 to 3.13) | 410.79 (136.66 to 798.49) | 0.98 (0.31 to 1.94) | 0.13 (0.04 to 0.25) | -2.15 (-2.31 to -2.00) |
| Shanxi | 237.55 (71.50 to 486.70) | 1.25 (0.33 to 2.64) | 596.89 (204.00 to 1153.70) | 1.32 (0.40 to 2.68) | 0.12 (0.04 to 0.23) | 0.63 (0.46 to 0.79) |
| Sichuan | 1138.65 (470.84 to 2027.56) | 1.49 (0.54 to 2.83) | 2122.45 (722.80 to 4073.85) | 1.65 (0.54 to 3.20) | 0.13 (0.05 to 0.25) | 0.73 (0.56 to 0.90) |
| Tianjin | 80.36 (29.60 to 147.68) | 1.20 (0.40 to 2.29) | 179.54 (52.82 to 356.06) | 0.85 (0.23 to 1.74) | 0.12 (0.03 to 0.22) | -1.10 (-1.22 to -0.98) |
| Tibet | 69.83 (35.21 to 115.95) | 4.88 (2.30 to 8.30) | 103.43 (49.82 to 171.01) | 4.08 (1.78 to 7.10) | 0.18 (0.08 to 0.30) | -0.76 (-0.93 to -0.59) |
| Xinjiang | 184.18 (72.64 to 332.41) | 2.23 (0.78 to 4.17) | 683.23 (305.30 to 1175.00) | 2.99 (1.19 to 5.38) | 0.16 (0.07 to 0.28) | 1.56 (1.35 to 1.78) |
| Yunnan | 544.86 (219.72 to 998.13) | 2.32 (0.84 to 4.40) | 1303.56 (484.68 to 2410.04) | 2.48 (0.85 to 4.72) | 0.13 (0.05 to 0.25) | 0.62 (0.46 to 0.77) |
| Zhejiang | 715.36 (378.57 to 1149.02) | 2.24 (1.13 to 3.68) | 1312.92 (585.40 to 2216.00) | 1.54 (0.67 to 2.66) | 0.19 (0.09 to 0.32) | -1.10 (-1.24 to - 0.96) |

ASMR=age-standardized mortality rate; PAF=population attributable fraction; EAPC=estimated annual percentage change.
