# Supplementary material for: The burden of chronic kidney disease attributable to high sodium intake: a longitudinal study in 1990–2019 in China
Source: Front Nutr. 2025 Jan 15;11:1531358. doi: 10.3389/fnut.2024.1531358 (PMC11783680; doi:10.3389/fnut.2024.1531358)
Supplement: Supplementary file 2 [file Table_2.DOCX]

***Supplementary Tables 2*.** DALYs and ASDR of chronic kidney disease attributable to high sodium intake in 1990 and 2019 and the temporal trends from 1990 to 2019.

| **Characteristics** | **1990** | | **2019** | | | | **1990-2019** |
| --- | --- | --- | --- | --- | --- | --- | --- |
|  | **DALYs,**  **No. (95% UI)** | **ASDR per**  **100,000 No. (95% UI)** | **DALYs,**  **No. (95% UI)** | **ASDR per**  **100,000 No. (95% UI)** | | **PAFs %**  **(95% UI)** | **EAPC (%) in**  **ASDR No. (95%CI)** |
| **Region** | | | | | | | |
| Anhui | 27734.58 (15170.59 to 43046.35) | 63.22 (33.16 to 99.56) | 40623.38 (19362.66 to 67606.68) | 44.04 (20.92 to 73.13) | 0.18 (0.09 to 0.28) | | -0.73 (-0.92 to -0.53) |
| Beijing | 6235.29 (3375.77 to  9600.16) | 61.77 (32.63 to 95.71) | 10744.66 (4693.87 to 1  8195.95) | 31.24 (13.53 to 53.23) | 0.15 (0.07 to 0.26) | | -2.31 (-2.50 to -2.12) |
| Chongqing | 7363.94 (3244.18 to  12603.29) | 54.77 (23.1 to 95.45) | 20641.50 (8364.05 to  37020.51) | 48.99 (20.2 to 87.69) | 0.15 (0.06 to 0.25) | | 0.27 (0.03 to 0.51) |
| Fujian | 13258.12 (6233.19 to 22106.64) | 60.74 (27.43 to 102.39) | 21525.09 (9189.2 to  37260.14) | 40.78 (17 to 71.33) | 0.15 (0.06 to 0.25) | | -1.05 (-1.21 to -0.89) |
| Gansu | 4426.04 (902.83 to  9527.10) | 27.53 (5.14 to 61.34) | 9987.85 (2235.88 to  20921.49) | 28.20 (6.14 to 59.96) | 0.08 (0.02 to 0.18) | | 0.67 (0.48 to 0.86) |
| Guangdong | 25437.12 (9838.10 to 46360.43) | 51.84 (19.15 to 95.44) | 45445.82 (16872.70 to 84689.93) | 32.40 (11.48 to 60.92) | 0.12 (0.04 to 0.22) | | -1.29 (-1.58 to -1.00) |
| Guangxi | 15741.75 (5500.47 to 29175.73) | 50.71 (16.68 to 95.11) | 26397.84 (6796.56 to  54226.31) | 41.97 (10.51 to 87.11) | 0.10 (0.02 to 0.19) | | -0.09 (-0.33 to 0.14) |
| Guizhou | 20582.66 (10021.09 to 33937.01) | 89.12 (42.24 to 147.75) | 28236.41 (11497.00 to 50105.01) | 63.41 (25.54 to 113.25) | 0.14 (0.06 to 0.25) | | -0.94 (-1.08 to -0.80) |
| Hainan | 2059.52 (730.41 to  3940.06) | 42.46 (14.33 to 83.13) | 4151.29 (1225.27 to  8181.96) | 35.34 (9.89 to 70.57) | 0.10 (0.03 to 0.20) | | -0.20 (-0.36 to -0.04) |
| Hebei | 29415.76 (15334.46 to 46830.33) | 58.78 (29.51 to 95.67) | 58691.29 (26311.8 to  98833.95) | 55.97 (24.50 to 95.29) | 0.15 (0.07 to 0.25) | | 0.31 (0.13 to 0.50) |
| Heilongjiang | 13392.72 (6174.75 to 22576.80) | 54.33 (23.4 to 94.44) | 20672.08 (7444.62 to  38491.69) | 31.88 (11.03 to 60.27) | 0.12 (0.04 to 0.22) | | -1.40 (-1.60 to -1.20) |
| Henan | 29002.63 (14854.94 to 46428.47) | 43.1 (21.32 to 70.4) | 52385.11 (23953.20 to 88514.45) | 40.98 (18.50 to 70.24) | 0.16 (0.07 to 0.26) | | 0.63 (0.40 to 0.86) |
| Hong Kong | 3775.32 (1678.70 to  6270.92) | 65.34 (28.21 to 109.79) | 5972.21 (2239.03 to  11287.44) | 42.71 (16.55 to 79.61) | 0.14 (0.06 to 0.25) | | -1.08 (-1.29 to -0.88) |
| Hubei | 27564.97 (13824.40 to 44240.08) | 65.01 (30.95 to 107.25) | 44167.62 (17467.28 to 78001.08) | 49.96 (19.2 to 89.4) | 0.14 (0.06 to 0.24) | | -0.45 (-0.61 to -0.29) |
| Hunan | 37927.16 (17612.33 to 63995.07) | 75.64 (33.37 to 130.8) | 79096.73 (31821.50 to 138397.68) | 79.34 (30.63 to 140.24) | 0.15 (0.06 to 0.25) | | 0.72 (0.59 to 0.86) |
| Inner Mongolia | 9137.16 (4717.94 to  14712.50) | 60.55 (29.49 to 99.46) | 18629.79 (9170.82 to  30169.76) | 49.52 (23.43 to 81.35) | 0.17 (0.08 to 0.27) | | -0.28 (-0.45 to -0.10) |
| Jiangsu | 31817.88 (17770.21 to 49187.99) | 116.91 (64.11 to 182.93) | 37784.59 (17970.28 to 62389.10) | 63.95 (29.57 to 106.63) | 0.17 (0.08 to 0.28) | | -0.72 (-0.89 to -0.54) |
| Jiangxi | 25385.66 (12397.97 to 41976.15) | 42.87 (19.99 to 71.93) | 41690.17 (17814.12 to 73255.28) | 31.13 (13.57 to 54.09) | 0.15 (0.07 to 0.25) | | -1.66 (-1.96 to -1.36) |
| Jilin | 16249.73 (8879.78 to 25316.78) | 87.87 (46.24 to 138.68) | 22202.94 (9706.04 to  37945.50) | 48.90 (20.95 to 84.41) | 0.14 (0.06 to 0.25) | | -1.61 (-1.85 to -1.36) |
| Liaoning | 14163.52 (5597.29 to 25609.32) | 43.6 (16.35 to 81.3) | 30250.20 (11815.44 to 54372.47) | 38.20 (14.61 to 69.27) | 0.13 (0.05 to 0.23) | | 0.16 (-0.12 to 0.45) |
| Macao | 167.85 (77.93 to  280.15) | 58.92 (26.74 to 99.21) | 362.13 (154.52 to  619.91) | 36.51 (14.93 to 63.31) | 0.15 (0.07 to 0.26) | | -1.20 (-1.44 to -0.96) |
| Ningxia | 974.73 (317.68 to  1870.55) | 34.4 (10.15 to 67.85) | 2773.77 (978.87 to  5187.19) | 33.86 (11.27 to 65.43) | 0.11 (0.04 to 0.21) | | 0.41 (0.19 to 0.62) |
| Qinghai | 2498.53 (1326.65 to  3931.94) | 86.96 (44.19 to 138.22) | 5763.86 (2859.83 to  9346.44) | 81.25 (38.4 to 135.08) | 0.17 (0.08 to 0.27) | | 0.09 (-0.04 to 0.22) |
| Shaanxi | 17816.18 (8616.55 to 29057.59) | 70.71 (32.37 to 116.86) | 30870.57 (14207.79 to 51554.98) | 55.42 (25.25 to 93.73) | 0.16 (0.08 to 0.27) | | -0.34 (-0.49 to -0.20) |
| Shandong | 32408.09 (16492.18 to 52153.69) | 46.05 (22.8 to 75.57) | 54004.04 (25507.30 to 90677.99) | 34.87 (16.33 to 58.83) | 0.16 (0.08 to 0.26) | | -0.35 (-0.69 to -0.01) |
| Shanghai | 7665.72 (3634.92 to  12461.13) | 50.98 (23.28 to 84.73) | 13125.80 (5663.80 to  22870.86) | 30.01 (12.78 to 52.92) | 0.14 (0.06 to 0.25) | | -1.79 (-2.00 to -1.58) |
| Shanxi | 8529.37 (3015.4 to  16564.59) | 37.85 (12.46 to 75.25) | 20164.26 (8172.85 to  35985.95) | 39.38 (15.23 to 72.06) | 0.13 (0.05 to 0.23) | | 0.69 (0.54 to 0.84) |
| Sichuan | 41539.75 (18757.02 to 71608.77) | 45.82 (19.77 to 80.8) | 62349.63 (25141.15 to 111429.63) | 47.00 (18.93 to 84.22) | 0.14 (0.06 to 0.24) | | 0.56 (0.40 to 0.73) |
| Tianjin | 2928.03 (1214.14 to  5223.91) | 36.43 (14.46 to 65.28) | 6629.34 (2238.52 to  12228.71) | 28.86 (9.48 to 54.30) | 0.12 (0.04 to 0.22) | | -0.28 (-0.47 to -0.10) |
| Tibet | 2414.54 (1262.98 to  3907.30) | 148.9 (76.52 to 242.1) | 3361.86 (1778.05 to  5265.50) | 108.93 (54.32 to 176.25) | 0.18 (0.10 to 0.28) | | -1.24 (-1.43 to -1.04) |
| Xinjiang | 7271.71 (3083.48 to  12656.02) | 71.61 (28.48 to 126.95) | 21979.68 (11015.90 to 35626.00) | 80.06 (38.28 to 134.20) | 0.17 (0.08 to 0.27) | | 0.74 (0.58 to 0.90) |
| Yunnan | 18440.03 (8076.91 to 32699.91) | 67.91 (28.51 to 122.43) | 38798.28 (16396.45 to 67663.59) | 65.74 (26.97 to 116.07) | 0.14 (0.06 to 0.25) | | 0.33 (0.20 to 0.46) |
| Zhejiang | 24999.99 (14264.27 to 38193.01) | 67.63 (37.71 to 104.5) | 42663.28 (22463.61 to 66727.23) | 47.20 (24.55 to 74.49) | 0.20 (0.11 to 0.31) | | -0.79 (-0.98 to -0.59) |

DALYs=disability-adjusted life years; ASDR=age-standardized DALYs rate; PAF=population attributable fraction; EAPC=estimated annual percentage change.
